# 1297. Azithromycin vs Beta Lactams in Acute Exacerbations of COPD

**DOI:** 10.1093/ofid/ofab466.1489

**Published:** 2021-12-04

**Authors:** Pramodini Kale-Pradhan, Nour Baalbaki, Bianca Aprilliano, Christopher Giuliano, Carrie Hartner, Leonard B Johnson

**Affiliations:** 1 Wayne State University/Ascension St. John Hospital, Detroit, Michigan; 2 Ascension St John Hospital, Detroit, Michigan; 3 Wayne State Unversity, Detroit, Michigan

## Abstract

**Background:**

Bacterial infections cause approximately 50% of Acute Exacerbations of COPD (AECOPD). Current guidelines recommend a wide range of antibiotics, but evidence comparing agents is limited. The purpose of this study is to compare the effectiveness of azithromycin to beta lactams in the treatment of hospitalized patients with AECOPD.

**Methods:**

A multicenter, retrospective, observational study of adult patients admitted with AECOPD who received at least two consecutive days of either a beta lactam or azithromycin were included. The primary endpoint was treatment failure which is a composite endpoint defined as in-hospital mortality, admission to intensive care, initiation of invasive mechanical ventilation, requirement of a new antibiotic, steroid therapy escalation, or readmission due to AECOPD within 30 days. Secondary endpoints included each individual component of the composite endpoint and length of stay.

**Results:**

Of 11,395 patients screened, 595 met the inclusion criteria (428 were treated with azithromycin and 167 patients were treated with a beta lactam). The most common reason for exclusion was the receipt of both azithromycin and beta-lactam in 9857 patients. The patients were similar except the azithromycin group was more likely to be African-American and less likely to have failed an outpatient antibiotic. Treatment failure rate was 19.6% in the azithromycin group and 32.3% in the beta lactam group (P=0.001). Patients in the beta lactam group were more likely to experience in-hospital mortality (P=0.023), require a new antibiotic during admission (P< 0.001), and were more likely to be readmitted within 30 days of discharge due to AECOPD (P=0.032). Length of stay was significantly shorter in the azithromycin group compared to the beta lactam group. There were no statistically significant differences in the rates of adverse events among both groups.

**Conclusion:**

Treatment failure rate and length of stay were significantly higher in the beta lactam group compared to the azithromycin group. However, there were no differences in the side effect profile among both groups. Further studies should be performed to confirm these findings.

**Disclosures:**

**All Authors**: No reported disclosures

